# Fresh and hardened properties of alkali-activated slag concrete: The effect of fly ash as a supplementary precursor

**DOI:** 10.1016/j.jclepro.2022.133362

**Published:** 2022-10-10

**Authors:** Yubo Sun, Zhiyuan Liu, Saeid Ghorbani, Guang Ye, Geert De Schutter

**Affiliations:** a Magnel-Vandepitte Laboratory, Department of Structural Engineering and Building Materials, Ghent University, 9052, Ghent, Belgium; b Microlab, Section of Materials and Environment, Faculty of Civil Engineering and Geosciences, Delft University of Technology, Stevinweg 1, 2628, CN Delft, the Netherlands

**Keywords:** Alkali-activated material concrete, Blast furnace slag, Coal fly ash, Rheology, Strength development

## Abstract

The present study explores the possibility of replacing blast furnace slag (BFS) with coal fly ash (FA) to produce alkali-activated material (AAM) concrete with hybrid precursors. With an increased FA replacement ratio, the reaction kinetics, fresh and hardened properties of AAM mixtures have been investigated. The retardation effect on the reaction kinetics with an increased FA content has been observed, which not only extended the induction period along with the heat flow evolution but also reduced the cumulative heat release up to 24 h. Spherical FA particles can provide a ball-bearing effect to improve the workability of the hybrid AAM mixtures, while FA also slows down the deterioration of fresh properties since they are less reactive compared to BFS particles. Regarding the strength development, FA results in the reduction at all curing ages in the mixtures with a low silicate modulus (Ms0.25). Similarly, reduction in 1-day compressive strength has been detected in high silicate modulus mixtures (Ms0.5) with FA replacement, while the mixture with 10% FA exhibits the highest compressive strength among Ms0.5 concretes at later curing ages. Bigger capillary pores have been detected in AAM mixtures with an increase in FA content. However, AAM with 10% FA shows the lowest porosity in Ms0.5 mixtures, which is in agreement with the compressive strength results.

## 1 Introduction

As the mainstream precursors in alkali-activated materials (AAMs), investigations on ground granulated blast furnace slag (BFS) and coal fly ash (FA) have been intensively performed to better understand the reaction process. The microstructure of reaction products in AAMs is highly dependent on the availability of calcium content in the precursors (Provis et al., 2015), and thus the reaction proceeds with different mechanisms. Starting from the chemical composition of precursors (Provis and Van Deventer, 2013), AAMs could be divided into low-Ca and high-Ca systems, with FA (Class F according to ASTM C618) and BFS being the most representative precursors, respectively. In low-calcium systems, the main reaction product is alkali aluminosilicate (N-A-S-H) gel (Provis and Van Deventer, 2013; Provis et al., 2005), which has a highly crosslinked, disordered pseudo-zeolitic structure. While in the case of a high-calcium binder, calcium aluminosilicate hydrate (C-A-S-H) gels with a tobermorite-like structure are predominant (Provis and Van Deventer, 2013). However, the C-A-S-H gel could be significantly different from the major hydration product, i.e. C–S–H in Portland cement (Myers et al., 2013; Puertas et al., 2011), since the low Ca/Si ratio and high Al content result in more cross-linking between the dreierketten chains of the tobermorite-like gel (Myers et al., 2013, 2015). Besides, in hybrid systems containing both high and low-calcium precursors, the co-existence of such gels in the reaction product has been detected (Bernal et al., 2011; Ismail et al., 2014; Yip et al., 2005). However, it's been proposed that there's a tendency towards a C-A-S-H type of gel in the final products, which is more stable at high alkalinity (Yip et al., 2005; Garcia-Lodeiro et al., 2011).

Out of the reaction mechanism, the inherent properties of precursors also further affect both fresh and hardened properties of AAMs mixtures. Given the highly amorphous and reactive nature of BFS (Ben Haha et al., 2011; Kim et al., 2013), previous studies confirmed the promising mechanical strength (Fernández-Jiménez et al., 1999; Collins and Sanjayan, 1999; Wang et al., 1994) and durability (Chi, 2012; Aydın and Baradan, 2014) of alkali-activated slag (AAS) mixtures. However, in the fresh state, BFS-based AAM mixtures exhibited short setting time, fast slump loss, and high structural build-up rate (Jang et al., 2014; Dai et al., 2020; Lu et al., 2021) when solely used as the precursor. The workability deterioration is ascribed to the accumulation of primary C-(A)-S-H gels (Palomo et al., 2014; Palacios et al., 2021; Palacios and Puertas, 2011), which is formed due to the interaction between the calcium cations dissolved from the slag particles and the silicate ions from the activators. In the case of FA-based AAMs, in general, an elevated curing temperature of 60–85 °C is required to facilitate the activation process (Zhang et al., 2020), as the reactivity of FA is insufficient at ambient temperatures (Puertas et al., 2000; Somna et al., 2011). FA-based AAMs after high-temperature curing exhibited desired mechanical and long-term properties (Zhang et al., 2020; Chindaprasirt et al., 2007; Swanepoel and Strydom, 2002; Panias et al., 2007).

The hybrid AAM system has been taken into great consideration, where BFS and FA are blended as the precursor, to reach complementary advantages and compensate for the defects mentioned above. It has been reported that the increment in FA content could improve the workability of AAM mixtures (Ishwarya et al., 2019; Arbi et al., 2015; Nath and Sarker, 2014). Yang et al. (2018) proposed the fluidizing effect of fly ash microsphere, which effectively reduces the internal fractions between solid particles and mitigates the agglomeration of flocs to release the free water. Dai et al. (2020) addressed that an increase in FA content extended the setting time and slowed down the structural buildup of hybrid AAMs. The studies from Gao et al. (2016) revealed that the addition of FA into BFS can effectively reduce the shrinkage of hardened samples. On the other hand, it is suggested that the mechanical properties of FA-based AAMs in ambient curing conditions could be improved with the incorporation of BFS (Nath and Sarker, 2014; Puligilla and Mondal, 2013; Deb et al., 2014), which is attributed to the free calcium ions. Thus, a dense microstructure is formed due to the co-existence of C-A-S-H and N-A-S-H gels (Ismail and El-Hassan, 2018; Guo and Pan, 2018). Therefore, it is beneficial to blend BFS and FA as a hybrid AAM mixture regarding both fresh and hardened properties.

Furthermore, as an industrial by-product from coal-fired power plants generated during the combustion of pulverized coal (Yao et al., 2015; Blissett and Rowson, 2012), the availability of FA as supplementary cementitious material or precursor in AAMs is directly correlated to the production level of power plants. On the pathway to meet EU greenhouse gas (GHG) emission targets (Meinshausen et al., 2009; Alola et al., 2021; Akdag and Yıldırım, 2020), a coal phase-out policy has been proposed (Akerboom et al., 2020; Keles and Yilmaz, 2020; Fermeglia et al., 2020; Anke et al., 2020) to terminate the coal-based power production and expand the renewable energy sources. Coal-fired power plants have been planned to shut down across European countries, which will dramatically reduce the supply of FA in future decades. Consequently, the role of FA in AAM productions will be converted from the priority into a supplementary precursor, especially in EU countries.

This study aims to investigate the influence of FA to substitute BFS in AAM concrete with an increased replacement ratio. Rheometry has been performed on AAM concrete mixtures to evaluate their fresh properties and better understand the rheology evolution of AAM concrete with hybrid precursors, which is seldom published in the literature. Besides, the effects of partial replacement with FA on the strength development of AAM concrete have been studied as well. The results obtained also provide guidance on the mix design of hybrid AAM concrete with desired fresh and hardened properties.

## 2 Experimental method

### 2.1 Materials

**Fig. 1:**
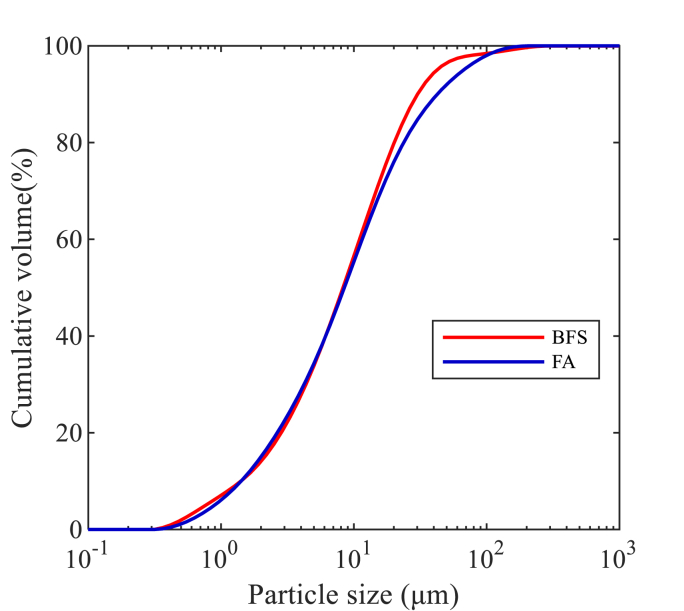
Particle size distribution of BFS and FA by laser diffraction.

**Fig. 2:**
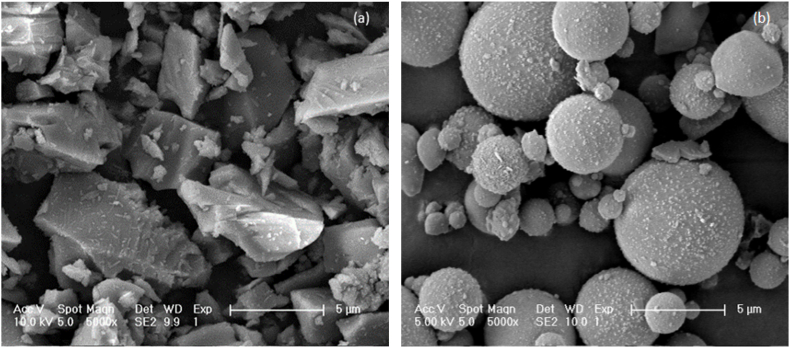
Morphology by SEM (5000 × magnification) (a) BFS particles; (b) FA particles.

The BFS as a major precursor in this study was provided by Ecocem Benelux B.V., with a density of 2890 $\text{kg}/{\text{m}}^{3}$. The low-calcium (Class F) fly ash was produced for use as type II filler in cementitious materials according to NEN-EN 450-1: 2012, and it was provided by Vliegasunie B.V., with a 2300 ± 200 $\text{kg}/{\text{m}}^{3}$ particle density. The BFS and FA used as the precursors in this study have similar particle size distributions detected by laser diffraction as presented in Fig. 1, with $d_{50}$ values of 8.28 and 8.48 μm, respectively. Disparate features on surface morphology were visualized with a scanning electron microscope (SEM), as shown in Fig. 2. Angular particles with irregular shapes have been observed in BFS samples, while FA particles were spherically shaped with various diameters. Details of their chemical compositions determined by X-ray fluorescence (XRF) and loss on ignition (LOI) are listed in Table 1.

**Table 1:** Chemical composition of BFS/FA measured by XRF and LOI (mass %).

Precursor	CaO	${\text{SiO}}_{2}$	${\text{Al}}_{2}{\text{O}}_{3}$	MgO	${\text{SO}}_{3}$	${\text{TiO}}_{2}$	${\text{K}}_{2}\text{O}$	${\text{Fe}}_{2}{\text{O}}_{3}$	MnO	${\text{ZrO}}_{2}$	Other	LOI a
BFS	40.9	31.1	13.7	9.16	2.31	1.26	0.69	0.40	0.31	0.12	0.05	0.10
FA	3.74	56.7	24.0	1.75	1.04	1.16	2.30	6.34	0.06	0.10	2.81	2.86

a LOI measured by TG analysis at 950 °C.

Sodium hydroxide and sodium silicate were combined as the hybrid activator in this study. Reagent-grade sodium hydroxide anhydrous pearls (>99%) were provided by Brenntag N.V., and the sodium silicate solution (15% Na_2_O, 30% SiO_2_, and 55% water) was provided by PQ Corporation.

River sand and gravel were used as the fine and coarse aggregate to produce AAM concretes in this study, and they were air-dried before mixing. The physical properties of aggregates are given in Table 2.

**Table 2:** Physical properties of aggregates.

Aggregate	Sand 0–4 mm	Coarse 2–8 mm	Coarse 8–16 mm
Specific gravity	2.65	2.64	2.67
Water absorption (%)	0.33	0.65	0.55

### 2.2 Mixture proportions

In this study, two AAM concretes with BFS as the sole precursor were designed as the reference mixtures (F1 and F5 in Table 3). In both mixtures, the sodium concentration was fixed at 4% in the activators (Zhang et al., 2021), while the silicate modulus (Ms) was designed at two different levels (Ms of 0.25 and 0.5, respectively). Moreover, the reactive content including all precursors and solid portions in the activator was kept as a constant of 391 kg/m^3^. Water content was counted as the sum of the water fraction from sodium silicate solutions and the externally added water. The water to binder (w/b) ratio was fixed at 0.45 in all AAM concretes. Aggregate packing was designed to reach between A16 and B16 curves indicated in DIN 1045-2, and the aggregate to paste mass ratio was kept constant at 3.2. Furthermore, with a constant w/b ratio of 0.45, the aggregate to paste volume ratio was also fixed at 2.2 in AAM concretes. The estimation has been made that the air content of BFS-based AAM concrete is 1% (Provis et al., 2019). By using substitute precursors, the BFS content in each reference mixture was replaced with FA at 10%, 20%, and 40% mass ratios. Other fractions excluding the precursors were kept unchanged to prepare AAM concretes. Details of mix designs are presented in Table 3. The alkaline activators were prepared by dissolving the alkaline and silicate compounds in tap water 24 h before mixing.

**Table 3:** Mixture proportion of AAS concretes.

Mix	Precursors			Activators				Reactive content (kg/m^3^) d	Extra water (kg/m^3^)	w/b e	w/b* f	A/P g	Aggregate (kg/m^3^) h		
	BFS (kg/m^3^)	FA (kg/m^3^)	FA a Ratio	Sodium hydroxide (kg/m^3^)	Sodium silicate (kg/m^3^)	Na_2_O b	Ms c						0–4 mm	2–8 mm	8–16 mm
F1	369	0	0	16.66	12.30	4%	0.25	391	169.26	0.45	0.43	3.2	726	501	588
F2	332.1	36.9	10%										726	501	588
F3	295.2	73.8	20%										726	501	588
F4	221.4	147.6	40%										726	501	588
F5	366	0	0	14.17	24.40		0.5		162.58				726	501	588
F6	329.4	36.6	10%										726	501	588
F7	292.8	73.2	20%										726	501	588
F8	219.6	146.4	40%										726	501	588

a Defined as the mass proportion of FA in precursors.

b Represented as the mass percentage of BFS.

c Defined as the molar ratio between SiO_2_ and Na_2_O in the activator.

d Defined as the total mass of precursors and solid activators.

e Defined as water content in both aqueous activator and extra water added separately divided by the sum of precursor and solid activators.

f Indicating the actual w/b ratio in the concrete mixture by excluding the aggregate water absorption.

g Defined as the mass ratio between aggregate and AAS paste.

h Designed to reach between A16 and B16 curves indicated in DIN 1045-2.

### 2.3 Testing program

#### 2.3.1 Isothermal calorimetry

Calorimetry studies were performed on the paste fractions in each AAM concrete to investigate the reaction kinetics of AAM mixtures. The heat flow evolution and cumulative heat release along the activation process were detected by TAMAIR isothermal calorimeter. To prepare the AAM pastes, solid precursors and activator solutions were manually mixed in a plastic cup for 2 min until a homogeneous mixture. Subsequently, 14 ± 0.01 g of AAS paste was loaded into a glass ampoule immediately and sealed with a plastic lid. The ampoules were then loaded into the isothermal channels in the calorimeter, and the exothermic behavior of AAM pastes was recorded at 20± 0.5 °C for 24 h. Results of heat flow evolution and cumulative heat were normalized into each gram of reactive content in the binder (including precursors and solid activators).

#### 2.3.2 Concrete mixing

**Fig. 3:**
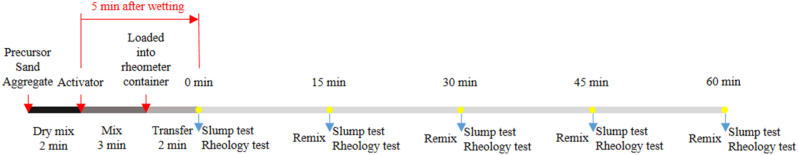
Mixing and testing protocol for fresh AAM concrete mixtures.

The mixing protocol of AAM concretes is illustrated in Fig. 3. All solid components (precursors and aggregates) were loaded into a planetary mixer and dry blended for 2 min. Subsequently, the activator solutions were added into the mixture and mixed for another 3 min. Fresh AAM concrete mixtures were used for the following tests on fresh and hardened properties.

#### 2.3.3 Test on fresh properties

Slump and rheological tests were performed simultaneously on ready-mixed AAM concretes to study their fresh properties. As illustrated in Fig. 3, the tests were repeated every 15 min in the first hour to investigate the workability retention properties. In this study, ‘0 min’ was defined as the moment when the first group of tests began, which was actually 5 min after the wetting of precursors. In addition, a 1-min remix was performed on the AAM concrete mixtures with a handheld mixer before each group of tests. The slump test was conducted with an Abrams cone according to EN 12350-2. Stress growth tests were carried out to determine the static yield stress, while the dynamic yield stress and plastic viscosity values were derived from flow curve tests.

**Fig. 4:**
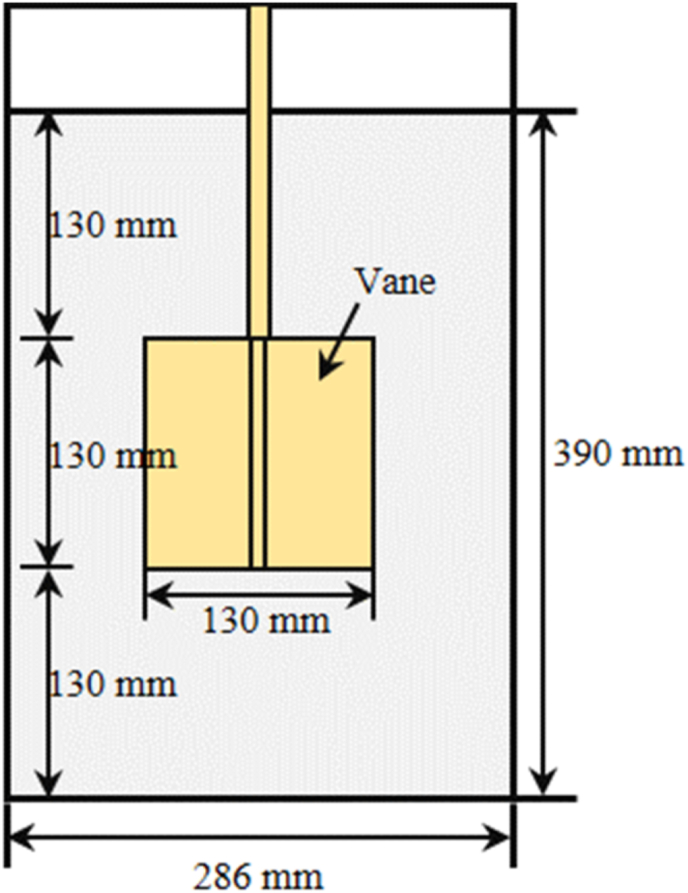
Geometry of ICAR Plus rheometer.

Rheology tests were performed with ICAR Plus rheometer fitted with a 4-blade vane of which the geometry of the longitudinal cross-section is shown in Fig. 4. The flow curves of different AAS mixtures were obtained using the down-ramp portion of the Torque-Rotational speed result determined by the rheometer. Bingham model was applied to fit the linear relationship presented in the flow curves. The Reiner-Riwlin equation (Feys et al., 2013) for Bingham model was applied to derive the dynamic yield stress (Pa) and plastic viscosity (Pa·s) from the torque-rotational speed relationship. For each AAS mixture, the experiments were performed twice at a target testing age, and the average value was reported in this study.

**Fig. 5:**
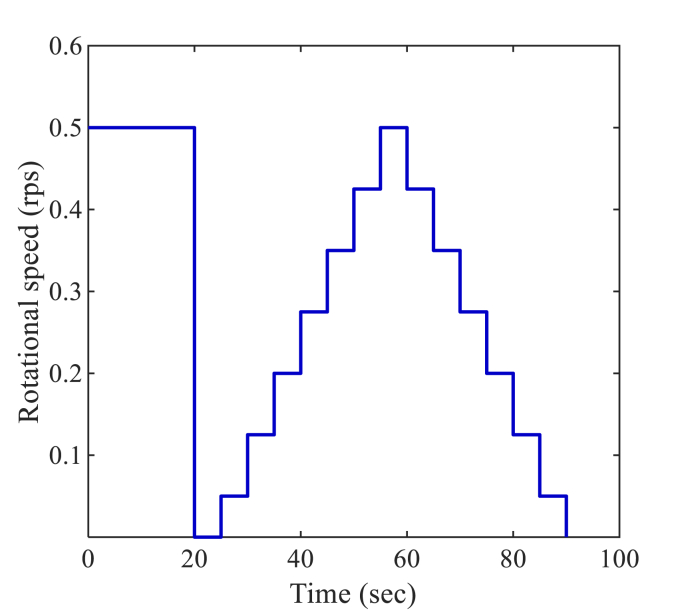
Shear protocol used in flow curve test.
• The fresh concrete was then recollected into the container and left at rest for the subsequent tests.
• Finally, the concrete was remixed in the container with a handheld mixer for 1 min before the next group of tests.

The tests were conducted with the following steps to keep the shear history consistent (Ferraris et al., 2001; Yahia, 2014) among different AAM concretes:

• The cylindrical rheometer container was filled with around 20 L fresh concrete. Subsequently, the rheometer vane was immersed into the concrete.
• The static yield stress was determined by the stress growth test. A constant rotational speed of 0.025 rev/s was applied until a peak torque occurred, which was recorded to derive the static yield stress.
• Afterwards, the flow curve test was conducted with the shear protocol illustrated in Fig. 5. A pre-shear corresponding to a rotational speed of 0.5 rev/s was applied for 20 s. The flow curve test was subsequently initiated with a stepwise increasing rotational speed. For each step, the measurement was conducted for 5 s, and the torque values were recorded to derive rheological parameters.

#### 2.3.4 Compressive strength

The AAS concrete mixtures after fresh property tests were cast into 100 mm size cubic molds to determine the strength development. Hardened concrete samples were demolded after 24 h and sealed in a plastic bag. The specimens were placed in a curing chamber at 20 °C with 95% relative humidity. Compressive strength tests were performed at 1, 7, 28, and 91 days according to EN 12390-3.

#### 2.3.5 Mercury intrusion porosimetry

Mercury intrusion porosimetry (MIP) tests were carried out on the hardened AAM paste samples to study the properties of pore structures. The remaining paste prepared for calorimetry studies was cast into plastic molds and cured at 20 °C under a sealed condition. After 28 days, the hardened AAM pastes were demolded and split into smaller pieces (approximately 4–5 mm^3^) for later MIP measurements. A solvent replacement with 50/50 (vol) methanol/acetone solutions was conducted to terminate the activation reaction (Chen et al., 2014). Afterwards, the AAM samples were vacuum dried for another 7 days before testing. MIP tests were conducted with a ThermoFischer Scientific Pascal 140 & 440 series mercury intrusion porosimeter, with a maximum pressure of 200 MPa. The contact angle between the mercury and the specimen surface was 140° and the surface tension was 0.48 N/m (Zhang et al., 2010).

## 3 Results and discussion

### 3.1 Calorimetry

**Fig. 6:**
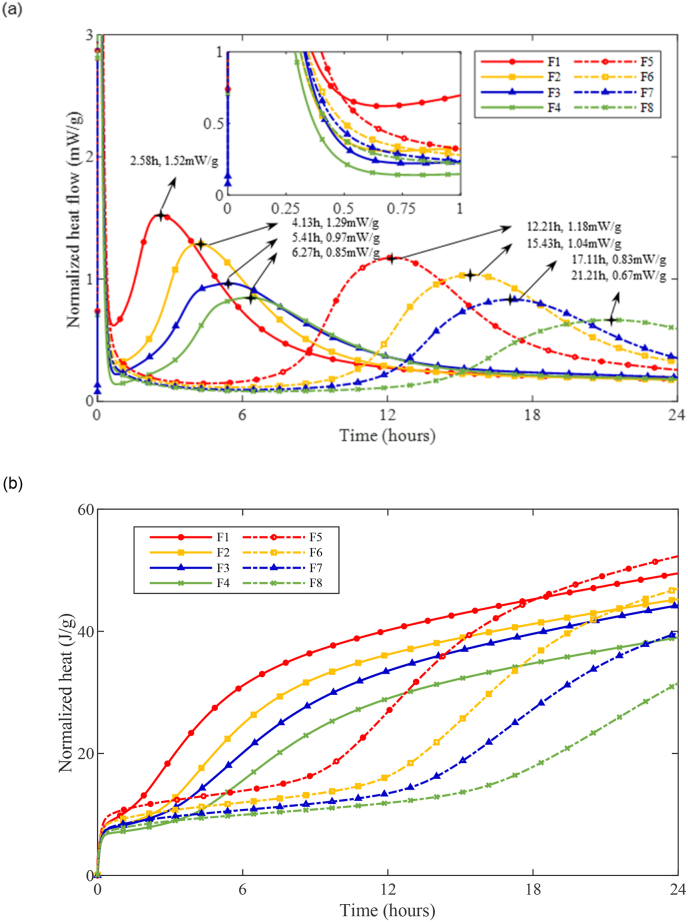
Heat evolution of AAM pastes (a) Normalized heat flow evolution; (b) Cumulative heat evolution.

The calorimetry results of AAM mixtures on the paste fraction are presented in Fig. 6. The reaction process of AAM mixtures could be divided into the following 5 stages according to the exothermic behavior: dissolution, induction, acceleration, deceleration, and the steady stage (Scrivener and Nonat, 2011).

Among Ms0.25 mixtures (solid curves indicated in Fig. 6), the reaction proceeded very fast that the induction period was seldom detected after dissolution. Followed by the initiation of the acceleration stage, which is accompanied by the formation of reaction products (Huanhai et al., 1993; Shi, 1995). In the AAM paste F1 (Fig. 6 (a)), a sharp growth in heat flow has been detected after the dissolution stage. The acceleration peak was detected at 2.58h with a maximum heat flow of 1.52 mW/g. By replacing BFS with 10% FA in F2, the exothermic behavior after dissolution turned moderate, and the acceleration peak progressively became broader and less intensive. Comparing to F1, the acceleration peak was delayed by 1.55 h in F2, while the maximum heat flow was declined by 15.1%. It is indicated that the substitution with FA resulted in a retardation effect on the reaction kinetics, which might be attributed to the less reactivity of FA (Puertas et al., 2000; Somna et al., 2011). Such retardation became more evident at higher replacement ratios that the acceleration peak in F3 and F4 was further postponed till 5.41 and 6.27 h, with a reduction in the maximum heat flow of 36.2% and 44.1% compared to F1, respectively. Moreover, the replacement with FA also reduced the total amount of 1-day heat release as shown in Fig. 6 (b). With 100% BFS as the precursor, a cumulative heat of 49.5 J/g was detected in F1. By replacing BFS with 10%, 20%, and 40% FA, the cumulative heat was dropped by 8.5%, 10.5%, and 21.2%, respectively.

In Ms0.5 mixtures (dashed curves in Fig. 6), an obvious induction period occurred after dissolution. In addition, the maximum heat flow in F5 was delayed by 9.63 h comparing to F1, while the peak value in heat flow was also reduced by 22.4%. Therefore, the reaction was retarded by using higher Ms. The extra silicate species in the activator not only inhibited the dissolution of Si from precursor (Zuo and Ye, 2020) but also reduced the alkalinity of the activator solution (Chang, 2003), which both slowed down the dissolution of precursors and resulted in the retardation effect on the reaction kinetics. In the meantime, the FA replacement also further retarded the activation reactions in Ms0.5 mixtures. By replacing 10% BFS with FA, the acceleration peak was delayed by 3.22 h, while the maximum heat flow in the acceleration/deceleration period was also decreased by 11.9% in F6 compared to F5. Furthermore, the FA-induced retardation became more pronounced with higher replacement ratios. Two major increment stages were detected in the cumulative heat curves of Ms0.5 mixtures. The first increment was ascribed to the heat release during dissolution period, due to the wetting and dissolution of precursors (Huanhai et al., 1993; Ravikumar and Neithalath, 2012). It has been detected that the F5 with 100% BFS released the greatest amount of heat during the dissolution stage among Ms0.5 mixtures. However, the heat release during dissolution gradually reduced with higher FA replacement ratios, which indicates that BFS is more reactive than FA in the dissolution stage. Such observation also holds for Ms0.25 mixtures. Subsequently, the cumulative heat release in Ms0.5 mixtures reached a plateau, as the reaction came to the induction period. Eventually, the 24-h cumulative heat release in F6, F7, and F8 was reduced by 10.1%, 24.0%, and 39.9% compared to F5, respectively.

To sum up, the replacement of BFS with FA in AAM mixtures brought significant retardation on the reaction kinetics, due to the less reactivity and deficiency of calcium ions in FA (Kumar et al., 2010).

### 3.2 Fresh properties

**Fig. 7:**
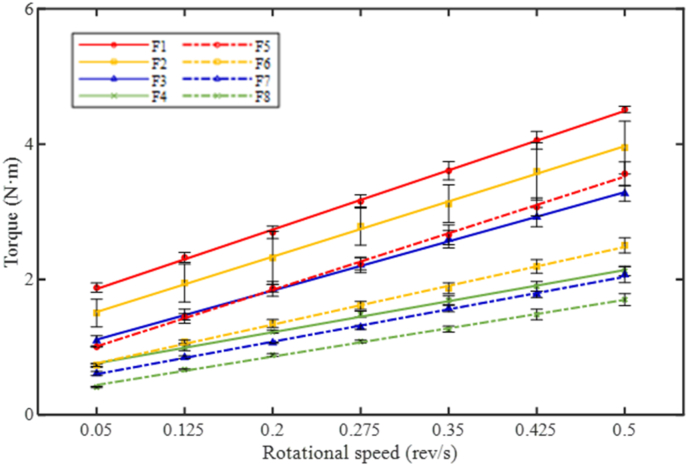
Flow curves of AAS concrete mixtures in Torque-Rotational speed relationship at 0 min.

Slump test and rheological test with the ICAR Plus rheometer were performed every 15 min in the first hour after mixing to characterize the fresh properties of AAM concrete mixtures. Static yield stress was determined by the stress growth test with the rheometer. Meanwhile, the flow curves of each mixture at target testing ages (for instance, flow curves at 0 min as shown in Fig. 7) were derived from the downward portions of the torque-rotational speed relationship. By applying the Bingham model, the dynamic yield stress and plastic viscosity were derived from Reiner-Riwlin equations.

#### 3.2.1 Initial fresh properties

**Fig. 8:**
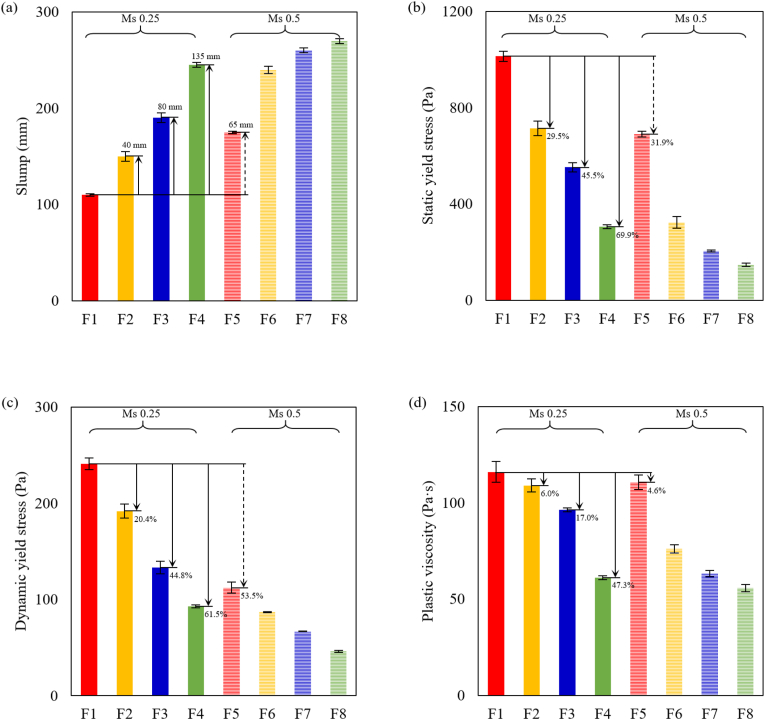
Initial fresh properties of AAM concretes (a) Slump value; (b) Static yield stress; (c) Dynamic yield stress; (d) Plastic viscosity.

The initial fresh properties of AAM concrete mixtures are displayed in Fig. 8. As shown in Fig. 8 (a), an initial slump value of 110 mm has been detected in the BFS-based concrete mixture F1. By replacing 10% BFS with FA, the slump value of F2 at 0 min was improved 40 mm compared to F1. With further increasing the FA content, the initial slump values increased by 80 and 135 mm with 20% and 40% replacement ratios, respectively. Similarly, the increase in slump values has been observed in Ms0.5 mixtures as well by improving the FA replacement ratio. The initial static yield stress of AAM concretes is presented in Fig. 8 (b). In both Ms0.25 and Ms0.5 groups, the static yield stress dramatically reduced with the inclusion of higher FA content. Comapring to F1, the static yield stress of F2, F3, and F4 at 0 min were reduced by 29.5%, 45.5%, and 69.9% respectively. Dynamic yield stress and plastic viscosity parameters at 0 min derived from flow curve measurements are presented in Fig. 8 (c) and (d). Significant reductions in both parameters have been observed with an increasing FA replacement ratio in AAM concrete mixtures.

The initial slump values and rheological parameters well illustrated the fluidizing effect due to the substitution with FA, which is attributed to several mechanisms. First of all, it has been addressed that FA contains a much smaller specific surface area than BFS (Nedeljković et al., 2018; Siddique and Khan, 2011). Therefore, replacement with FA reduced the water demand upon surface wetting of precursors after mixing to increase the free water content (Aboulayt et al., 2018). Furthermore, the FA particles with a spherical shape (see Fig. 2 (b)) can provide a “ball-bearing” effect (Yang et al., 2018; Chen et al., 2013) in fresh mixtures. With the spherical FA particles, the interparticle frictions between angular slag particles and solid aggregate grains are effectively reduced (Zhang et al., 2016a; Nanthagopalan et al., 2008). In addition, spherical FA particles also contribute to a lubricating effect (Yang et al., 2018), which assists to break down the interparticle connections between flocs and release the water trapped in agglomerations (Duan et al., 2017; Vance et al., 2013). Yang et al. (2018) also concluded that fly ash microsphere could be used as an inorganic dispersing agent to improve the workability of AAMs. Accordingly, the initial fresh properties of AAM concrete mixtures were significantly improved with an increasing FA replacement ratio.

Moreover, it should be aware that the initial slump values of F7 and F8 reached 260 and 270 mm, respectively. No bleeding or segregation has been observed in these mixtures. Further increase in FA replacement ratio did not result in much improvement on the initial workability and rheological parameters. It is also noteworthy Ms0.5 concrete mixtures in general exhibited better initial fresh properties compared to the corresponding Ms0.25 mixtures, which is ascribed to the fluidizing effect of extra silicate content (Alonso et al., 2017; Aydin and Baradan, 2014) and the reduction in solid concentration (Struble and Sun, 1995) in Ms0.5 mixtures.

#### 3.2.2 Workability retention properties

The workability retention properties of AAM concretes were characterized by the slump value and rheological parameters evolutions in the first hour.

**Fig. 9:**
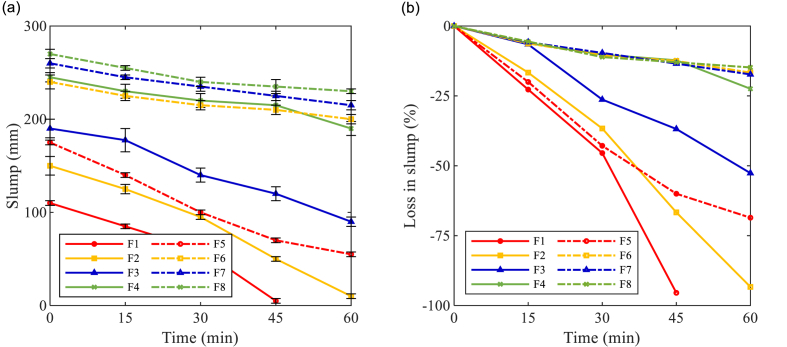
Results of slump tests against time (a) Slump values (b) Loss in slump.

As displayed in Fig. 9 (a), BFS-based concrete mixtures (F1 and F5) showed the lowest slump values over time in both Ms0.25 and Ms0.5 groups. By improving the FA replacement ratio, the slump curves gradually shifted upwards, which indicates the fluidizing effect of FA particles. Meanwhile, Ms0.5 mixtures (dashed curves in Fig. 9 (a)) also exhibited better workability than the corresponding Ms0.25 mixtures (solid curves in Fig. 9 (a)) in the first hour. Regarding the percentage of loss in slump values, as shown in Fig. 9 (b), the slump loss of Ms0.25 mixtures was much rapid than Ms0.5 mixtures at the same testing ages. Among Ms0.25 mixtures, the slump loss curves significantly turned less steep with the increase in FA replacement ratio. It is indicated that the structural build-up was slowed down due to the substitution with the less reactive FA (Puertas et al., 2000; Somna et al., 2011). An increase in slump loss slope has been observed in Ms0.25 mixtures between 30 and 45 min, ascribed to the early exothermic reaction process detected by the initiation of acceleration stage in calorimetry after 30 min (enlarged view in Fig. 6 (a)). In Ms0.5 mixtures, referring to F5 and F6, the slump loss curve was significantly flattened with only 10% FA. Such a great reduction in slump loss rate might be attributed to both the aforementioned FA- and silicate-induced retardation effect. Further increase in FA replacement ratio did not result in obvious changes in F7 and F8, but the slump loss maintained a low level. It is indicated that the solid particles were dispersed far apart enough from each other (Roussel et al., 2010), and the structural development was to a great extent slowed down.

**Fig. 10:**
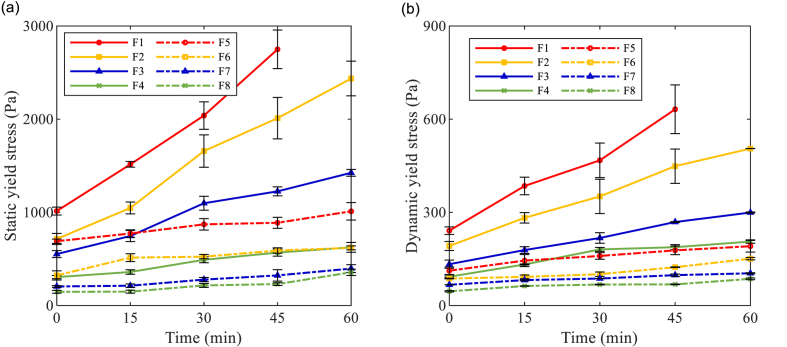
Yield stress evolution of AAM concretes against time (a) Static yield stress; (b) Dynamic yield stress.

The yield stress evolution curves of AAM concretes are presented in Fig. 10. It's been observed that the static and dynamic yield stress evolution almost follows the same trend. In F1, the static yield stress was increased by 49.4% between 0 and 15 min. While during the same period, 10%, 20%, and 40% replacement with FA resulted in 46.6%, 35.8%, and 18.1% increment in static yield stress in corresponding mixtures, respectively. With the increase in FA replacement ratio, the yield stress curves progressively moved downwards and became less steep. The result further illustrates the fluidizing and retardation effects of FA particles, which reduced the yield stress and slowed down the structural development, respectively. The maximum values of yield stress were detected in F1 (100% BFS mixture with Ms0.25) with time elapsed. A rapid growth in yield stress was observed in F1 and it became no longer workable after 45 min. Besides, Ms0.5 AAM concretes generally showed lower static and dynamic yield stress over time than Ms0.25 mixtures, due to the reduction in solid concentration by using higher Ms. Meanwhile, the slopes of yield stress curves in Ms0.5 mixtures are smaller than those of Ms0.25 curves. The results reveal that the extra silicate content in the activator also retarded the structural development in AAM mixtures. It is noteworthy that nearly identical static yield stress evolution has been detected between F4 and F6, while F6 exhibited much lower dynamic yield stress than F4 along with time. Despite the difference in FA content, it is indicated that the structural development in Ms0.5 mixtures is more reversible and easier to be broken down by the pre-shear applied before flow curve tests.

**Fig. 11:**
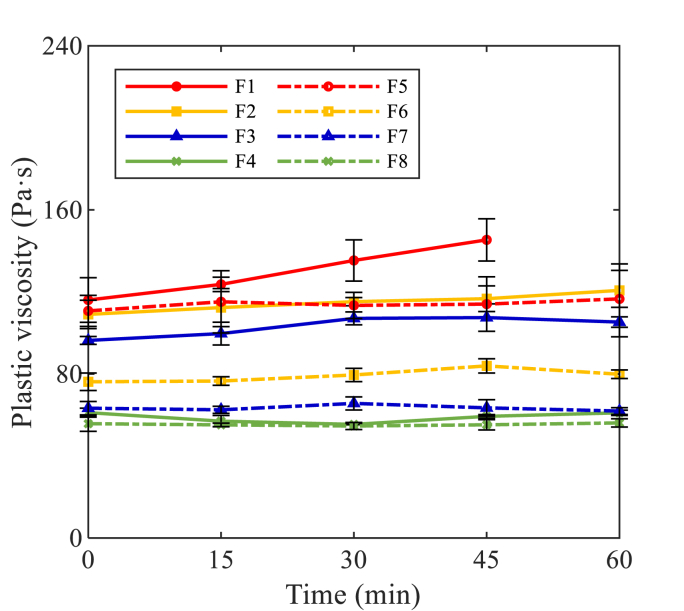
Plastic viscosity of AAM concretes against time.

The plastic viscosity of AAM concretes is shown in Fig. 11. The majority of AAM concrete mixtures kept a relatively constant viscosity within the first hour. The highest plastic viscosity values were observed in F1 (100% BFS, Ms0.25), with slight growth up to 45 min until the mixture lost its fluidity. Lower plastic viscosity has been detected in Ms0.5 mixtures compared to Ms0.25 mixtures, which indicates that the solid grains were more dispersed by the extra silicate content to reduce the interaction between solid particles (Vance et al., 2013). In both groups, the plastic viscosity of AAM mixtures gradually reduced with an increased FA replacement ratio, which is attributed to the dispersing effect of FA particles.

Accordingly, replacing BFS with FA improved the initial fresh properties and workability retention properties of AAM concrete mixtures. Due to the ball-bearing effect and less specific surface area of FA, more free water is released into the AAM mixture. In that case, the interactions between solid grains are mitigated and thus the workability of AAM mixtures is improved. Meanwhile, the FA content also leads to the retardation effect on the reaction kinetics due to less reactivity compared to BFS. Therefore, the FA replacement also slowed down the reaction process to improve the workability retention properties of AAM concrete mixtures. Besides, the results of this study also indicate that extra silicate content in the activator has similar effects as FA to improve the workability of AAMs.

### 3.3 Compressive strength development

**Fig. 12:**
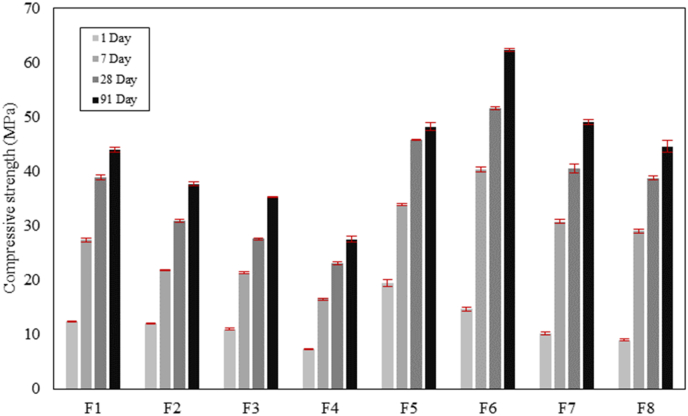
Compressive strength development of AAM concretes.

The compressive strength of AAM concrete at 1, 7, 28, and 91 curing days are presented in Fig. 12. In Ms0.25 concrete mixtures (F1, F2, F3, and F4), the inclusion of FA inhibited the strength development at all curing ages. The 1- and 91-day compressive strength of 12.5 and 44.0 MPa were detected in reference 100% BFS concrete F1, respectively. By replacing BFS with 10% FA, the compressive strength of F2 at corresponding curing ages was declined by 3.3% and 14.3%, respectively. With a higher FA content in F3 and F4, the compressive strength was further reduced, and the ultimate strength of F4 (40% FA) was decreased by 37.5% compared to F1.

Different strength development trends have been observed among Ms0.5 concrete mixtures (F5, F6, F7, and F8). Comparing to the reference mixture F5, the 1-day compressive strength was reduced by 25.0%, 47.8%, and 54.0% with 10%, 20%, and 40% FA replacement, respectively. However, the FA content facilitated the long-term strength development. Comparing to the 28-day results, the 91-day compressive strength of F6, F7, and F8 was increased by 17.1%, 17.4%, and 13.2%, respectively. While the strength in F5 only increased 5.1% during the same period. It is noteworthy that F7 and F8 almost reached equivalent compressive strength as F5 after 91 days, while the strength of F6 at 7, 28, and 91 days already exceeded the strength detected in F5.

Despite the less reactive nature and lower calcium content of FA comparing to BFS, the enhancement of strength development with FA replacement detected in Ms0.5 concretes might be ascribed to different mechanisms. Dong et al. (2020) reported lower strength in 100% BFS AAM mixtures compared to those with FA substitutions, and they interpreted the strength reduction by increased viscosity in the mixture, which entrapped more air bubbles during mixing. Moreover, spherical FA particles result in better compatibility with irregular-shaped particles (Liu et al., 2019), which promotes a denser packing (Aboulayt et al., 2018; Chapman, 1999) in the concrete. Other researchers also proposed that FA particles may provide extra nucleation sites for the reaction products (Palomo et al., 2014; Gao et al., 2015) which makes the microstructure even denser and contribute to higher strength. The pore structures of hardened AAM samples were investigated by MIP to further explore their strength development, which will be discussed in section 3.4.

### 3.4 Pore structures

**Fig. 13:**
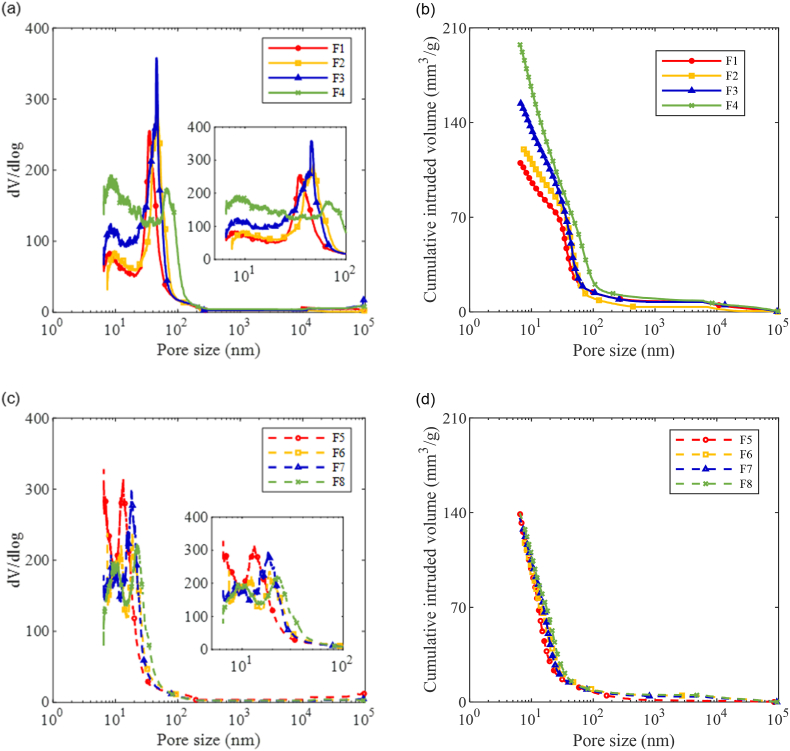
Pore structure of hardened AAM paste at 28 day (a) Pore size distribution of Ms0.25 AAM pastes; (b) Cumulative pore volume of Ms0.25 AAM pastes; (c) Pore size distribution of Ms0.5 AAM pastes; (d) Cumulative pore volume of Ms0.5 AAM pastes.

The pore structures of AAM pastes at 28 days are shown in Fig. 13, which are categorized into gel pores and capillary pores (Ma et al., 2013; Zhang et al., 2016b).

In Ms0.25 mixtures, as presented in Fig. 13 (a) and (b), the capillary porosity (around 20–100 nm) is slightly increased when the FA content is increased from 0 to 10%. With 20% FA replacement, the increase in gel porosity (<20 nm) is observed in conjunction with the increased capillary porosity. Furthermore, the total porosity of AAM paste drastically increased when the FA replacement ratio raised to 40%, especially in the fraction representing the gel pores. A rightward shift of the peak value in capillary pores has been observed as well in F4, which reveals a coarser pore size distribution. Therefore, the higher porosity and coarser pore size distribution associated with an increased FA replacement ratio result in the reduction in the compressive strength in Ms0.25 AAM concretes.

As shown in Fig. 13 (c) and (d), the FA replacement ratio seems to have a greater influence on the pore size distribution among Ms0.5 AAM mixtures. The peak referring to the gel pores occurred at around 10 nm in mixtures with FA replacement, while that of the F5 (without FA) is smaller than 10 nm. It is noteworthy that the peak representing gel pores is incomplete in F5. The results indicate that F5 may still contain a certain amount of smaller gel pores below 6.5 nm, which can not be measured with a maximum intrusion pressure of 200 MPa in this study. On the other hand, the capillary pore size was progressively enlarged with an increased FA replacement ratio. The results further illustrate the less reactivity of FA compared to BFS, which results in fewer reaction products to fill in the capillary pores between precursor grains. Nevertheless, the porosity of F6 is slightly lower than all other mixtures (see Table 4), which is in line with the 28-day compressive strength of AAM concrete samples. Thus, the increase in long-term strength development with FA replacement can be attributed to their denser microstructures (Liu et al., 2019; Chapman, 1999).

**Table 4:** Cumulative porosity of the hardened AAS pastes at 28 days.

Mixture	F1	F2	F3	F4	F5	F6	F7	F8
Cumulative porosity (%)	15.97	17.08	17.81	21.72	13.58	13.49	14.12	15.74

However, the increase in long-term strength development with FA replacement was not observed in Ms0.25 mixtures. It is indicated that sufficient silicate content is demanded to enhance the strength development with FA replacement. Fig. 13 shows a smaller capillary pore size of Ms0.5 mixtures (10–45 nm) compared to Ms0.25 mixtures (20–150 nm), which is attributed to the extra nucleation sites provided by the silicate content in the activator (Gebregziabiher et al., 2015). Given the redundant nucleation sites and reaction products to fill in the majority of capillary pores in Ms0.5 mixtures, the replacement with 10% FA resulted in an even denser microstructure due to the filler effect and compatibility with irregular-shaped particles (Liu et al., 2019; Deschner et al., 2012). In the case of Ms0.25 mixtures and Ms0.5 mixtures with a higher FA content, it is likely that the reaction products are not sufficient to fill in the capillary pores induced by FA replacement, and thus results in a more porous microstructure. Accordingly, the highest 28-day compressive strength was detected in F6.

**Fig. 14:**
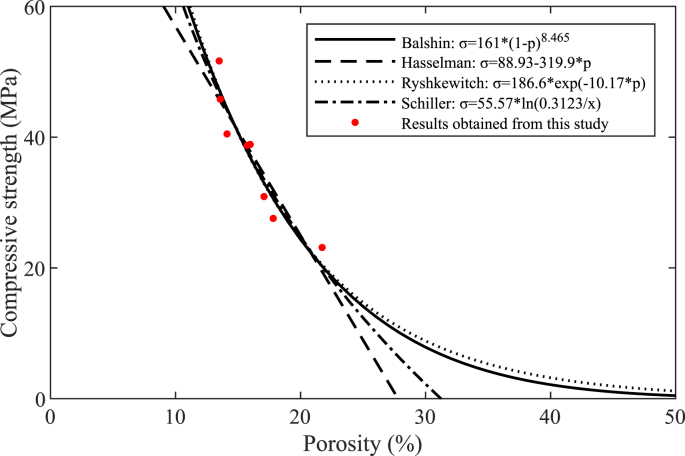
Strength-porosity relationship of AAM mixtures.

Great efforts have been made in previous studies to establish the strength-porosity relationship of porous materials. Four empirical models have been proposed to describe such relationships in concrete (Balshin, 1949; Hasselman, 1963; Ryshkewitch, 1953; Cristelo et al., 2013), as summarized in Table 5. These fitting curves are plotted in Fig. 14 to evaluate the strength and porosity parameters. A good correlation between results obtained from this study and empirical models has been derived to explore the strength-porosity relationship of AAM concretes. The results indicate that the empirical models are capable to predict the strength-porosity relationship of AAMs, which can provide guidance information on the packing of materials towards a target strength in AAMs.

**Table 5:** Empirical equations to describe the strength-porosity relationship.

Equations *	Empirical constants	References
$\sigma =\sigma_{0}{\left(1-p\right)}^{b}$	b	Balshin (Balshin, 1949)
$\sigma =\sigma_{0}-cp$	c	Hasselman (Hasselman, 1963)
$\sigma =\sigma_{0}e^{-kp}$	k	Ryshkewitch (Ryshkewitch, 1953)
$\sigma =n\;\ln\left(\frac{p_{0}}{p}\right)$	n	Schiller (Cristelo et al., 2013)

* σ is the compressive strength of the material, p is the porosity of the material, $\sigma_{0}$ is the theoretical strength at zero porosity, $p_{0}$ is the theoretical porosity at zero strength.

## 4 Conclusions

In this study, the coal fly ash (FA) is used as a substitute material to replace the blast furnace slag (BFS) to produce alkali-activated material (AAM) concrete with hybrid precursors. With an increased replacement ratio, the reaction kinetics, fresh and hardened properties of AAM mixtures have been investigated with different silicate modulus (Ms0.25 and Ms0.5), and the following conclusions can be drawn according to the results obtained:

• The replacement of BFS with FA induced a significant retardation effect on the reaction kinetics. The induction period in heat flow evolution is extended with an increase in FA replacement ratio, accompanied by the reduction of the maximum heat flow after dissolution. Meanwhile, the cumulative heat release up to 24 h of AAM paste is also decreased with higher FA replacement ratios.
• With the same FA replacement ratio, higher Ms values resulted in a significant extension in the induction period after dissolution. Meanwhile, the calorimetry results indicate that the maximum heat flow in Ms0.5 mixtures was reduced as compared to Ms0.25 mixtures.
• By increasing the FA content in both Ms0.25 and Ms0.5 mixtures, the initial workability in terms of slump values, yield stress, and plastic viscosity of AAM concrete is significantly improved, which is attributed to the spherical shape and ball-bearing effect of FA particles. Meanwhile, replacing BFS with FA also slows down the activation reaction to improve the workability retention properties of AAM concretes, as FA are less reactive in the alkaline environment compared to BFS.
• In Ms0.25 mixtures, the FA substitution reduces the compressive strength development at all curing ages, which becomes more pronounced at higher replacement ratios. In the case of Ms0.5 mixtures, the FA replacement results in the reduction of 1-day compressive strength, but facilitates the long-term strength development, especially with 10% FA.
• With an increase in FA content, higher porosity and bigger capillary pores have been detected in Ms0.25 mixtures, which confirms a more porous microstructure is formed. Similar increases in capillary pore size have been observed in Ms0.5 mixtures as well by improving the FA replacement ratio. However, the lowest porosity is detected in AAM with 10% FA, which is consistent with the maximum 28-day compressive strength results.
• The results from this study indicate that replacing a small portion of BFS with FA in a high Ms AAM mixture not only improves the workability but the long-term strength development is also promoted. However, an optimum FA content in a BFS-FA concrete regarding both fresh and hardened properties can be determined from trial mixes in specific applications, since the properties of precursors can be varied from one plant to another.

## CRediT authorship contribution statement

**Yubo Sun:** Conceptualization, Methodology, Investigation, Writing – original draft. **Zhiyuan Liu:** Conceptualization, Methodology, Writing – original draft. **Saeid Ghorbani:** Methodology, Writing – original draft. **Guang Ye:** Supervision, Writing – review & editing. **Geert De Schutter:** Funding acquisition, Supervision, Writing – review & editing.

## Declaration of competing interest

The authors declare that they have no known competing financial interests or personal relationships that could have appeared to influence the work reported in this paper.
